# High-Capacity Mesoporous Silica Nanocarriers of siRNA for Applications in Retinal Delivery

**DOI:** 10.3390/ijms24032753

**Published:** 2023-02-01

**Authors:** Amelia Ultimo, Mar Orzaez, Maria J. Santos-Martinez, Ramón Martínez-Máñez, María D. Marcos, Félix Sancenón, Eduardo Ruiz-Hernández

**Affiliations:** 1 School of Pharmacy and Pharmaceutical Sciences, Trinity College Dublin (TCD), D02 W272 Dublin, Ireland; 2 Centro de Investigación Príncipe Felipe, Eduardo Primo Yúfera 3, 46012 Valencia, Spain; 3 Unidad Mixta UPV-CIPF de Investigación en Mecanismos de Enfermedades y Nanomedicina, Universitat Politècnica de València, Centro de Investigación Príncipe Felipe, 46012 Valencia, Spain; 4 School of Medicine, Trinity College Dublin (TCD), D02 R590 Dublin, Ireland; 5 Trinity Biomedical Sciences Institute, Trinity College Dublin (TCD), D02 R590 Dublin, Ireland; 6 Instituto Interuniversitario de Reconocimiento Molecular y Desarrollo Tecnológico (IDM), Universitat Politècnica de València, Universitat de València, 46022 Valencia, Spain; 7 Unidad Mixta de Investigación en Nanomedicina y Sensores, Universitat Politècnica de València, Instituto de Investigación Sanitaria La Fe, 46026 Valencia, Spain; 8 CIBER de Bioingeniería, Biomateriales y Nanomedicina (CIBER-BBN), 28029 Madrid, Spain

**Keywords:** age-related macular degeneration, large pore mesoporous silica nanoparticles, siRNA delivery, VEGF silencing

## Abstract

The main cause of subretinal neovascularisation in wet age-related macular degeneration (AMD) is an abnormal expression in the retinal pigment epithelium (RPE) of the vascular endothelial growth factor (VEGF). Current approaches for the treatment of AMD present considerable issues that could be overcome by encapsulating anti-VEGF drugs in suitable nanocarriers, thus providing better penetration, higher retention times, and sustained release. In this work, the ability of large pore mesoporous silica nanoparticles (LP-MSNs) to transport and protect nucleic acid molecules is exploited to develop an innovative LP-MSN-based nanosystem for the topical administration of anti-VEGF siRNA molecules to RPE cells. siRNA is loaded into LP-MSN mesopores, while the external surface of the nanodevices is functionalised with polyethylenimine (PEI) chains that allow the controlled release of siRNA and promote endosomal escape to facilitate cytosolic delivery of the cargo. The successful results obtained for VEGF silencing in ARPE-19 RPE cells demonstrate that the designed nanodevice is suitable as an siRNA transporter.

## 1. Introduction

Nowadays, retinal vein occlusion, ocular tumours and several retinal diseases are known as the most common causes of progressive and irreversible angiogenesis-related blindness, i.e., age-related macular degeneration (AMD), in aged people, diabetic retinopathy in adults and premature retinopathy in children [1,2,3,4,5]. In particular, AMD is a multifactorial disease with a complex pathophysiology that leads to a progressive loss of central vision due to damaged retinal pigment epithelium (RPE) and photoreceptors in the macula, the central area of the retina [6]. AMD is responsible for more than half of the cases of legal blindness in elderly people in developed countries. An increased prevalence of the disease has been foreseen as a result of population ageing and underlines the urgency for implementing eye care therapies [7,8,9,10]. In its late stages, the disease can evolve into dry or wet AMD, the latter being the one that provokes a more severe vision loss [6,9]. Wet AMD occurs in patients who develop subretinal choroidal neovascularisation (CNV), an abnormal growth of the choriocapillaris through the membrane that separates the RPE from the choroid, the external highly vascularised layer which supplies the retina’s metabolic needs [11,12,13]. It has been demonstrated that persistent unregulated angiogenesis is mostly driven by an abnormal expression of the vascular endothelial growth factor (VEGF) in the RPE, the primary mediator of angiogenesis and vascular permeability which affects nearby tissues [7,14,15]. Therefore, VEGF represents the principal target of developed therapeutic strategies.

During the last decade, in addition to surgery, intravitreal injections of anti-VEGF monoclonal antibodies or gene therapy products have become the standard of care for CNV and have contributed to, and importantly reduced, AMD-related blindness [4,5,7,16,17]. Due to the complexity of the eye anatomy, intravitreal injections have been selected as the standard route of administration to reach the posterior eye segment. In fact, the physiological barriers that protect the eye from exogenous substances prevent an efficient delivery and sufficient bioavailability in the retina of those molecules when administered topically or systemically (<5% of drugs administered by drops and 1–2% of systemically administered doses reach the retina) [1,2,4,5,18,19,20]. Additionally, stem cell therapy has demonstrated the potential to regenerate the retina in patients, but presents challenges related to the obstruction of small vessels following injection, immune rejection, and the lack of control of the cell differentiation processes [21].

In 2004, Macugen^®^ (pegaptanib, by Bausch + Lomb/Pfizer), a pegylated aptamer, was the first anti-VEGF agent approved by the FDA for the treatment of wet AMD [16,22]. Later, Lucentis^®^ (ranibizumab, by Genentech/Novartis), a humanised antibody fragment that blocks the receptor binding domain of all isoforms of VEGF-A, and Eylea^®^ (aflibercept, by Regeneron/Bayer), a recombinant fusion protein with higher affinity for VEGF-A, became established treatments for wet AMD [16,23,24]. Beovu^®^ (brolucizumab, Novartis), a humanised single-chain antibody fragment with enhanced tissue penetration and increased binding affinity, is the most recent intravitreal anti-VEGF agent to receive FDA approval [25]. Nevertheless, despite the desirable therapeutic outcomes obtained, these approaches still present important issues, mainly due to their route of administration (i.e., intravitreal injections) and the need for frequent applications. Since these injections constitute a life-long medication for a chronic disease [14,19], the variety of side effects and risks related to reiterated administration (pain, infection, vitreous haemorrhage, intraocular inflammation, glaucoma, retinal damage or detachment, and cataract formation) also become a major hurdle for patient adherence. 

The ocular immune privilege is determined by physical barriers, the inhibitory microenvironment of immune-competent cells, and ocular active regulation of systemic immune responses that reduce the risk of inflammation [26,27]. Considering these factors and the low amount of therapeutics needed for eye treatment, the possibility of modulating protein expression by nanocarrier-assisted gene therapy represents an interesting alternative for the treatment of AMD. Thr first attempt of anti-VEGF sense oligonucleotide ocular delivery was carried out in 2004 by means of lipid-lysine dendrimers, with successful in vivo results for up to two months after treatment [28]. The integration of oligonucleotides in delivery systems facilitates cell penetration and allows protection against degradation that is needed for a sustained effect [29]. Currently, several clinical trials are being conducted for siRNA- or aptamer-based therapies against wet AMD [30,31,32,33]. 

For overcoming the major limitations associated with current therapeutic approaches for AMD, gene therapy can be combined with suitable nanovectors that improve its stability and provide a better penetration and accumulation in tissues with higher vascular permeability. By protecting drugs and therapeutic molecules from rapid degradation or excretion, the use of nanocarriers leads to higher retention times and facilitates a tuneable release [31,34,35,36]. Among the large assortment of available nanocarriers, mesoporous silica nanoparticles (MSNs) provide several advantages for the loading, protection, and delivery of biomolecules [37,38,39]. MSNs present tuneable pores with a high loading capacity, large surface area, a scalable synthesis, and the possibility of surface functionalisation for developing targeted or controlled release systems, as well as for increasing their bioavailability and cellular uptake [40,41,42,43,44,45,46]. In the case of large pore MSNs (LP-MSNs), the loading of nucleotides inside the pores can increase their protection [42]. Several MSN-based systems have been studied in vitro for ocular and retinal delivery [47,48,49,50,51]. In particular, pegylated and bevacizumab-loaded MSNs, administered by subconjunctival injection, have recently demonstrated the ability to almost totally inhibit retinal neovascularisation in vivo without toxic effects [51]. Actually, silicate nanoparticles themselves have been even suggested as inhibitors of VEGF-mediated retinal neovascularisation [12]. Further studies have shown how silica nanoparticle’s toxicity in the eye can be related to their size, and the ultrafine silica nanoparticles (≤40 nm) are the ones that exert toxicity to the cornea, both in vitro and in vivo, while sizes in the range of 50–150 nm are considered safe [52,53,54,55]. 

Based on the above, the main objective of the present work was the development of an LP-MSN-based nanosystem for the administration and controlled delivery of anti-VEGF siRNA molecules, and it’s in vitro assessment in retinal pigmented epithelial cells. LP-MSNs were synthesised, loaded with siRNA, and finally functionalised with polyethylenimine (PEI), which acts as a molecular gate for the controlled release of nucleic acid molecules while facilitating, as other cationic polyelectrolytes do [56], endosomal escape for cytosolic delivery. The obtained results of VEGF silencing in a human retinal pigment epithelial cell line (ARPE-19) highlight the noteworthy potential of the designed materials as siRNA carriers.

## 2. Results

### 2.1. Design and Synthesis of Functionalised LP-MSNs 

**Scheme 1:**
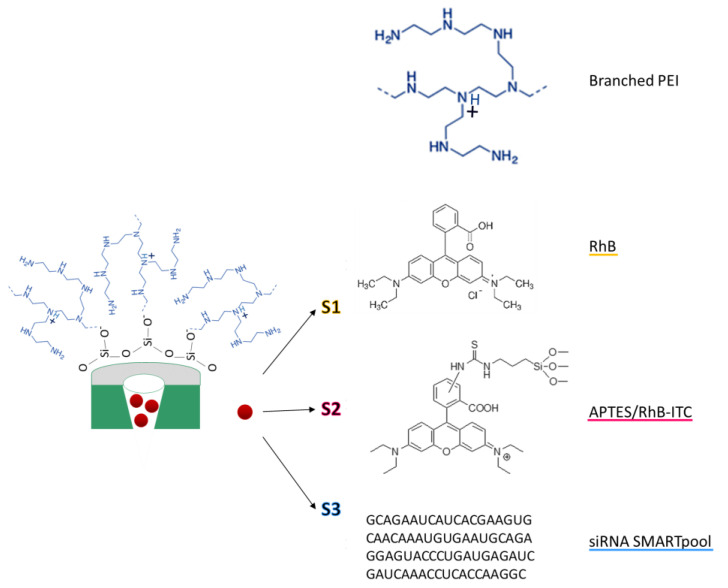
Schematic representation of the synthesised nanodevice. Rhodamine B (RhB), aminopropyltriethoxysilane (APTES) and rhodamine B isothiocyanate (RhB-ITC), or anti-VEGF siRNA were used to load or functionalise the large mesopores in the materials S1 (orange), S2 (pink) and S3 (blue), while the positively charged PEI chains are attached through electrostatic interactions to the negatively charged silica surface.

The main objective of this work was to develop gated materials with the ability to efficiently deliver siRNA molecules to RPE cells. LP-MSNs were synthesised in order to allow highly accessible internal spaces for siRNA molecule transport. Branched PEI 10 K was selected as the capping polymer for cargo-controlled release, which was anchored to the MSN surface through electrostatic interactions. This PEI with a molecular weight of 10 K was chosen as it has been demonstrated to have low cytotoxicity while keeping the ability to deliver nucleic acids to the cytoplasm [57]. Three different types of functionalised nanoparticles were developed (Scheme 1): S1, loaded with rhodamine B (RhB) dye and capped with PEI chains, which allowed performing the cargo release assay and verifying the PEI capping ability; S2, covalently functionalised with rhodamine B isothiocyanate (RhB-ITC) through 3-aminopropyltriethoxysilane (APTES) chains, and externally capped with PEI, employed to study nanoparticles cytotoxicity, cellular uptake, and hemocompatibility of the nanoparticles; and S3, the final solid loaded with anti-VEGFA siRNA and capped with PEI, developed for VEGF silencing in ARPE-19 cells. 

### 2.2. Nanoparticles Characterisation

**Figure 1:**
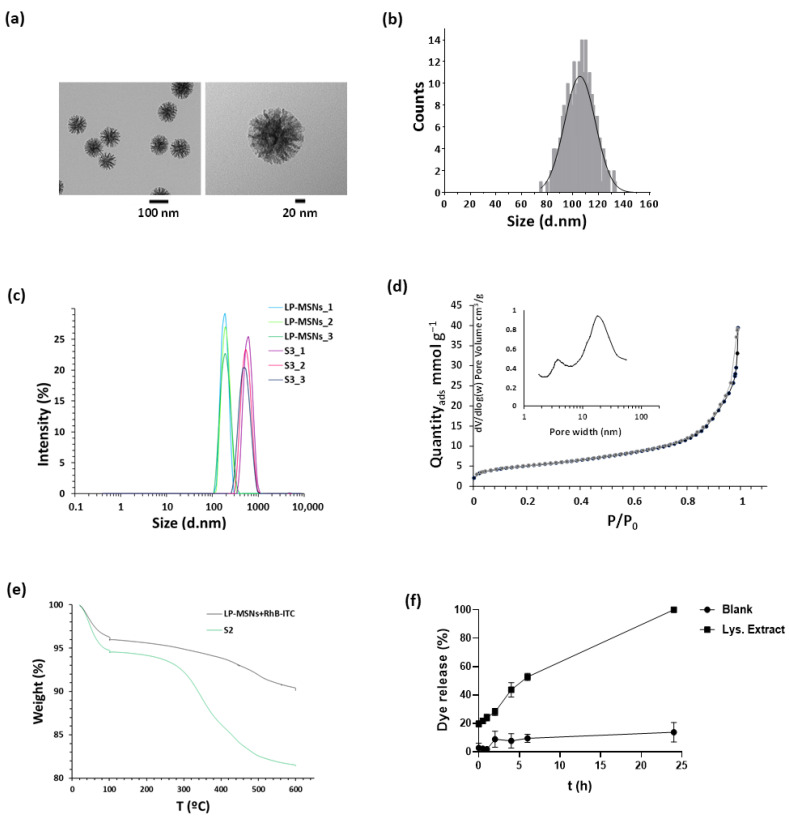
LP-MSN characterisation. (**a**) Representative transmission electron microscopy (TEM) images of LP-MSNs. (**b**) LP-MSN size distribution obtained by measurements taken from TEM images. (**c**) Dynamic light scattering (DLS) intensity distribution of LP-MSNs and S3 nanoparticles size. Three independent measurements for each type of nanomaterials are represented. (**d**) N_2_ adsorption–desorption isotherms for LP-MSNs. Inset: BJH pore size distribution. (**e**) Thermogravimetric analysis of LP-MSNs with anchored RhB-ITC and S2 nanoparticles. (**f**) Normalised release profiles of RhB from S1 nanoparticles in the absence or in the presence of purified lysosomal extract, obtained by measuring RhB fluorescence at 580 nm at different times.

Transmission electron microscopy (TEM) micrographs were taken to confirm nanoparticle size and morphology, as well as the pore structure of synthesised LP-MSNs. The representative images reported in Figure 1a show spherical monodisperse dendrimer-like nanoparticles with centre-radial large pores. An average size of 105 ± 12 nm was calculated from 300 measurements taken from TEM images (Figure 1b). The hydrodynamic size distribution of both LP-MSNs and S3 was also evaluated by dynamic light scattering (DLS) studies (Figure 1c). For LP-MSNs, the resulting hydrodynamic diameter was 198 ± 9 nm and it was 599 ± 99 nm for the S3 nanoparticles, with a polydispersity index of 0.23 and 0.31, respectively. 

The zeta potential of the starting LP-MSN nanoparticles was −40.4 mV, while after dye loading and functionalisation with PEI in S2, it increased to 27.2 mV due to the positive charge of the polymer. 

Figure 1d shows the obtained adsorption–desorption isotherm for LP-MSNs. The curve shows a type IV isotherm, typical of dendrimer-like mesoporous structures [58]. The Barrett–Joyner–Halenda (BJH) pore distribution graph indicates a broad distribution of pore size, with an average size of the main peak of 17 nm. The application of the Brunauer, Emmett, and Teller (BET) model resulted in a value of 398 m^2^ g^−1^ for the total specific surface area. 

The content of grafted RhB-ITC and capping PEI was determined by thermogravimetric analysis in S2 nanoparticles (Figure 1e), resulting, after water evaporation from the samples, in 6.4 mg of RhB-ITC and 7.7 mg of PEI per 100 mg of material. An amount of 45.6 μg/mg of siRNA loaded into S3 mesopores (43% of loading efficiency) was calculated, by the difference between the amount measured using a NanoDrop Spectrophotometer in the reaction mix and in the supernatant after nanoparticle separation by centrifugation. 

### 2.3. Cargo Release

The release of an entrapped guest molecule in response to given changes in the biological environment is an essential capacity of the gatekeeping mechanisms used during the development of stimuli-responsive mesoporous nanomaterials. Hence, dye release assays were carried out to check the gating ability of the synthesised materials. S1 nanoparticles loaded with RhB and externally functionalised with PEI were used to perform these studies. Purified lysosomal extract was used as release medium, as we speculate that cargo release will take place in the acidic environment of late endosomes and lysosomes, when proton sequestration by the polymer layer and the high ionic strength of the medium allows PEI chain detachment from the surface of the nanoparticles, leading to cargo delivery [59,60]. As observed in Figure 1f, when pH 7.4 phosphate buffered saline (PBS) was used as a blank medium, S1 nanoparticles only released a small amount of RhB in 24 h, indicating that most of the cargo was still protected inside the pores. In contrast, the presence of the lysosomal extract stimulated a significant increase in the release of the fluorescent dye as a function of time. These results confirm the release triggering mechanism of the nanodevice.

### 2.4. Nanoparticles Interaction and Activity in ARPE-19 Cells

**Figure 2:**
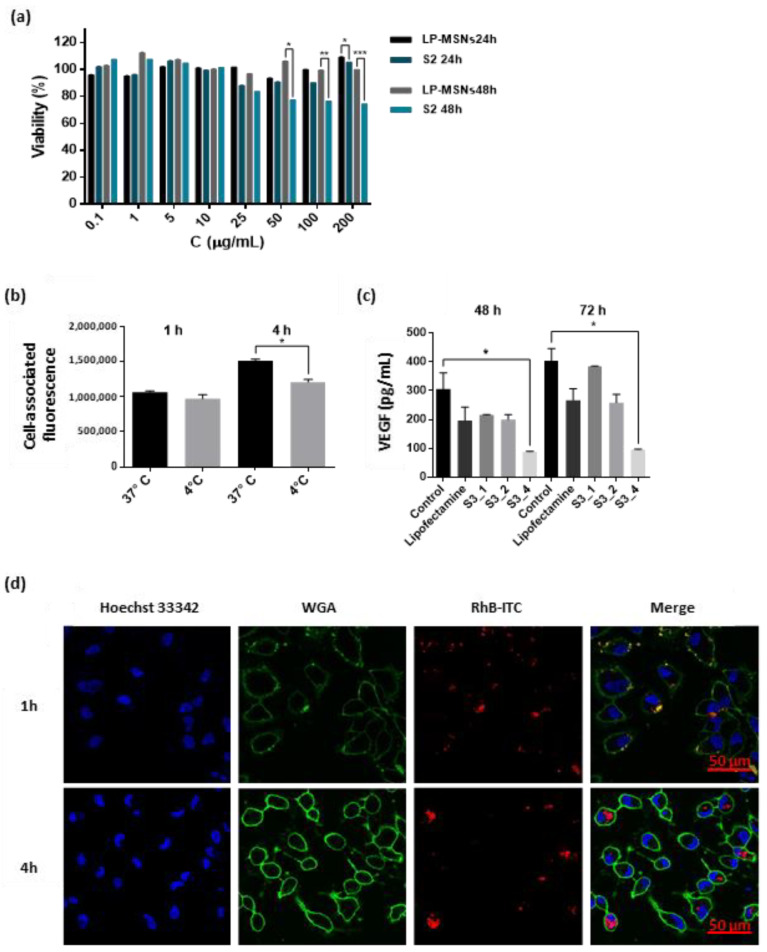
Biological validation of the developed nanomaterials. (**a**) ARPE-19 cell viability after 24 or 48 h of incubation with different concentrations of LP-MSNs or S2 nanoparticles. Plates were analysed by measuring absorbance at 490 nm after 1 h of further incubation with cell proliferation reagent WST-1. Data are expressed as means ± s.d. Asterisks indicate significant differences (* = *p* < 0.05, ** = *p* < 0.01, *** = *p* < 0.001) when the t-test was applied. (**b**) Cell-associated fluorescence over time of ARPE-19 cells treated with 50 μg/mL of S2 and incubated at 37 °C or 4 °C is represented. Data are expressed as means ± s.d. Asterisks indicate significant differences (*p* < 0.01) when the t-test was applied. (**c**) VEGF levels (pg/mL) in ARPE-19 cell supernatant after transfection with 100 nM siRNA/lipofectamine complex or treatment with 1× (S3_1), 2× (S3_2), or 4× (S3_4) the amount of siRNA in lipofectamine complex. Data are expressed as means ± s.d. Asterisks indicate significant differences (*p* < 0.05) when the t-test was applied. (**d**) Representative confocal microscopy images of ARPE-19 cells after 1 or 4 h of incubation at 37 °C with 50 μg/mL of S2. Cell nuclei are stained with Hoechst 33342 (blue) and cellular membrane with wheat germ agglutinin (WGA) (green). S2 nanoparticles are labelled with RhB-ITC (red).

Before proceeding to investigate the efficacy of the final nanoparticles in the cells of interest, the biocompatibility of S2 with ARPE-19 cells was tested. WST-1 viability tests at different times and concentrations of both bare (i.e., LP-MSNs) and functionalised (i.e., S2) nanoparticles were performed. As observed in Figure 2a, LP-MSNs showed no cytotoxicity even at the highest concentrations after up to 48 h of incubation, while S2 nanoparticles slightly decreased cell viability (to between 70 and 80% viability) after 48 h at concentrations equal to or higher than 50 μg/mL. In addition, S2 nanoparticle ability to enter ARPE-19 cells was confirmed by uptake studies monitored by both confocal microscopy and flow cytometry, tracking the RhB-ITC associated fluorescence. Confocal microscopy images demonstrate that nanoparticle uptake by ARPE-19 cells is a rapid process, since most of the nanoparticles in the images co-localise with the cellular membrane after 1 h, and almost all of them are inside the cytosol after 4 h (Figure 2d). Furthermore, the cell-associated fluorescence (CAF, resulting from percentage of positive cells × mean fluorescence/100) measured by flow cytometry indicated that, as it is quicker at 37 °C than at 4 °C during the first hours of incubation, cellular uptake is an energy-dependent mechanism [61,62], thus showing that nanoparticles are internalised by an endocytic pathway (Figure 2b).

Finally, the capacity of siRNA-loaded nanoparticles to silence VEGF in retinal pigmented epithelial cells was tested by incubating ARPE-19 cells with S3 nanoparticles at different concentrations, corresponding to 1×, 2×, or 4× the amount of siRNA in lipofectamine complex, used as positive control, for 6 h and analysing the expression of VEGF levels in the supernatant by ELISA 48 or 72 h after treatment. Untreated cells (negative control) and cells transfected with 100 nM siRNA/lipofectamine complex were also analysed with the same ELISA Human VEGF test. The obtained results (Figure 2c) showed that S3 represents a valid means for siRNA transport and delivery to the cells of interest. In particular, the highest amount of S3 used in the activity assays, corresponding to 4× the amount of siRNA/lipofectamine complex added to the plates, significantly reduced VEGF expression after both 48 and 72 h. In fact, a knockdown of up to 70% and 75% at 48 h and 72 h, respectively, after treatment of ARPE-19 with S3 nanoparticles was observed. Thus, the developed nanocarrier was demonstrated to provide siRNA protection, cellular penetration, endosomal escape, and consequent cytoplasmic release, confirming its potential as an effective method for nucleic acid delivery. 

### 2.5. Nanoparticles Interaction with Red Blood Cells and Platelets

With regard to the potential toxicity of the synthesised nanomaterials, a recent study in rats demonstrated that ocular topical administration of high concentrations (10 mg/mL) of 100 nm silica nanoparticles over a 12-week time span had no significant toxicity on hematologic, biochemical, or histopathologic parameters [54]. Likewise, MSNs demonstrated no toxic consequences in retinal endothelial and neural cells in vitro and in mouse retina in vivo [12,51]. 

**Figure 3:**
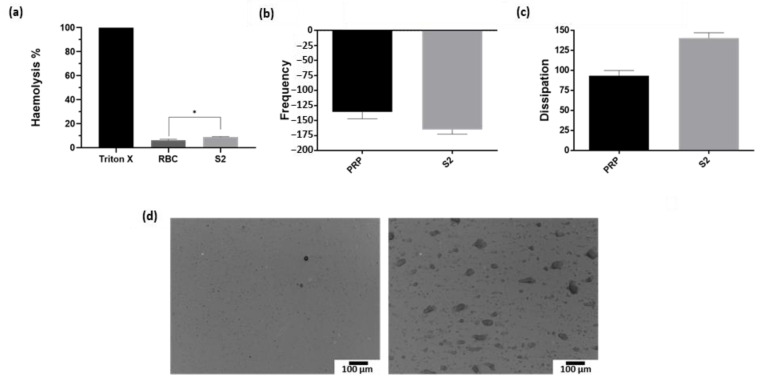
Haemocompatibility assessment of the developed nanomaterials. (**a**) Haemolysis induced by S2 nanoparticles compared to red blood cells (RBC) in the absence of nanoparticles. Triton X-100 was used as a positive control. Data are expressed as means ± s.d. Asterisks indicate significant differences (*p* < 0.05) when the t-test was applied. (**b**) Quantitative analysis of the effect of platelet-rich plasma (PRP) in the absence or presence of S2 nanoparticles on the frequency of the quartz crystal, as measured by the quartz crystal microbalance (QCM-D) (n = 2). (**c**) Quantitative analysis of the effect of PRP in the absence or presence of S2 nanoparticles on the energy dissipation, as measured by the QCM-D (n = 2). (**d**) Representative micrographs of the surface of fibrinogen-coated polystyrene-coated quartz crystals as viewed by optical microscopy, showing platelet aggregates following the perfusion of PRP in the absence (image on the left) or presence (image on the right) of S2 nanoparticles.

In our preliminary assessment, two different assays were performed to investigate the potential detrimental effect of PEI functionalised nanoparticles, S2, on red blood cells (haemolysis) and blood platelets. S2 nanoparticles at a concentration of 100 μg/mL, after 1 h of incubation with red blood cells, induced a haemolysis rate only slightly higher than the negative control without treatment (9% and 6%, respectively, Triton X-100 was used as a positive control and 100% haemolysis) (Figure 3a). S2’s effect on platelet aggregation was also studied using a quartz crystal microbalance (QCM-D). This is an extremely sensitive method capable of detecting microaggregation under flow conditions [63,64,65,66], and it has been previously used for investigating the interaction of various nanoparticles with blood platelets [67,68,69,70]. The principle of analysis of the QCM-D is based on the resonance frequency of a quartz crystal induced by applying an alternating electric field across the crystal. The device measures two parameters, the frequency of vibration (f) and energy dissipation (D), simultaneously and in real time. An increase in mass bound to the sensor surface causes the crystal oscillation frequency to decrease (negative f shift). If the layer deposited on the sensor surface is rigid and thin, the decrease in f is proportional to the mass [71]. However, if the layer attached to the sensor is thick and soft, measurements based on changes in f may result in underestimation of the deposited mass. In those situations, the analysis of the dissipation factor, related to the viscoelastic properties of the adsorbed layer, becomes crucial [72]. In fact, the perfusion of blood platelets through the QCM-D and their interaction with the surface of the sensors leads to the formation of a ‘viscoelastic layer’ as platelet aggregates deposit on the crystal. Therefore, when platelet function is investigated using QCM-D, more accurate measurements are obtained when both parameters (f and D) are investigated and analysed simultaneously [67,68,69,70]. As observed in Figure 3b, a larger decrease in crystal oscillation frequency was recorded during the perfusion of platelet-rich plasma (PRP) in the presence of 100 μg/mL of S2 nanoparticles, indicating an increase in mass bound to the quartz surface, namely, an increase in platelet aggregates. Consequently, an augmented energy dissipation was also observed under the same conditions (Figure 3c). In fact, the crystals’ micrographs taken by optical microscopy after the perfusion of PRP in the presence of S2 show the formation of some platelet aggregates on the surface of the sensor (Figure 3d). Even so, the effect of PEI-coated nanoparticles on platelet aggregation, probably due to the external PEI layer, can certainly be improved in future work by further functionalisation of the particle surface, for example, with polyethylene glycol (PEG) chains.

## 3. Discussion

In an AMD global burden projection for 2040, a high increase in the prevalence in people over 75 years in Europe and Oceania and a large spread of the disease in Asia have been foreseen due to population ageing [10]. Therefore, the development of innovative, more efficient, and safer therapeutic approaches for the treatment of the disease is an urgent medical challenge. Current treatments, although providing favourable results in CNV control, present several serious drawbacks. In fact, the estimated half-life of ranibizumab or aflibercept in the aqueous humour is less than five days, and monthly injections are required [73]. Furthermore, a recent deep review of 15 different randomised clinical trials that utilised ranibizumab and bevacizumab highlighted how the frequency of the anti-VEGF injections correlates directly with the improvement in patients’ visual acuity, and also demonstrated the existence of a ceiling effect after a given number of injections [74]. 

In this context, drug nanocarriers could represent an ideal means for enhancing drug bioavailability and, in particular, to facilitate ocular gene therapy. With the recent approval of Luxturna by the FDA, the first ocular gene replacement therapy to be directly administered for an inherited blinding eye condition [75], and there is a new enthusiasm among researchers about the development of new gene treatments for retinal diseases. Nevertheless, the potential of DNA or RNA administration is still constrained due to the lack of effective delivery systems that ensure appropriate protection, tissue penetration, and efficient and controlled release. A successful silicon-lipid nanoparticle approach for topical siRNA delivery to the anterior eye has been recently presented [76]. The developed systems, composed of silicon nanoparticles, lipids, and amino acids, with an siRNA content of between 30 and 115 µg/mg, produced a significant gene silencing effect in vivo with no adverse effects through non-invasive topical administration [76].

The results of the present work, instead, represent the first step in the development of LP-MSN-based nanodevices aimed to deliver siRNAs to RPE cells in the posterior eye. Regarding the nanocarrier size, it has already been shown that nanoparticles of 100–200 nm are preferable for ocular drug delivery, since they avoid clearance and increase circulation within the ocular fluidic barrier, while at the same time reducing the risk of irritation produced by larger particles [1,77]. Therefore, the synthesised dendrimer-like LP-MSNs with a diameter of around 105 nm and a high pore capacity could be an ideal platform for topical drug delivery to ocular tissues. 

As stated previously, several systems of nucleic acid nanocarriers for posterior eye delivery have been published. In particular, different studies have been carried out using poly(lactic-co-glycolic acid) (PLGA) nanoparticles for the delivery of anti-VEGF plasmids both intravitreally or systemically [30]. A successful attempt to target RPE cells by topical instillation instead was made in 2014 with transferrin targeted small liposomes loaded with plasmid DNA [78]. The employment of colloidal systems such as nanoparticles or liposomes that provide sustained release, indeed, as well as of penetration or viscosity enhancers, is the main approach to overcome the drawbacks related to the topical administration into the eye, namely the presence of the corneal and conjunctival barriers and lacrimal drainage [31,79]. Furthermore, the potential of tuneable nanoparticles, such as mesoporous silica nanoparticles, whose inner as well as outer surface can be remodelled at will, offers many possibilities for the optimisation of the charge and release of molecules for gene therapy. The inside of the pores can in fact be made more suitable for hosting siRNA molecules by functionalising them with positively charged compounds, such as APTES [80]. On the other side, external surface modification with polymers such as chitosan, PEG, or hyaluronic acid, or with ligands such as transferrin, albumin, folate, or RGD peptide, has shown to increase the delivery to the posterior chamber of the eye, representing promising design strategies for the development of new drug nanovehicles [77]. Such approaches are of special interest for the next stages of our investigation with the nanomaterials designed in this study.

In summary, the results obtained in this work indicate the potential of nanovehicles based on LP-MSNs for the sustained attenuation of VEGF in RPE cells by siRNA delivery. The developed system takes advantage of the high capacity and ease of access of the large pores of the mesoporous network to load and transport siRNA molecules, and of the capacity of PEI chains to promote endosomal escape to reach cytoplasmic delivery. The ability of the siRNA-loaded material, S3, to enter retinal cells, effectively deliver its cargo, and largely reduce the expression of VEGF in ARPE-19 cells has been demonstrated. Further optimisation of the system and in vivo studies of the impact on visual function, as well as of nanoparticle biodegradation and biodistribution, will be necessary to obtain a clinically acceptable product that could have high medical, social, and economic relevance in the treatment of non-curable retinal diseases.

## 4. Materials and Methods

### 4.1. Reagents

The chemicals cetyltrimethylammonium p-toluenesulfonate (CTATos), triethanolamine (TEA), tetraethylorthosilicate (TEOS), APTES, RhB, RhB-ITC, Lysosome Isolation Kit (LYSISO1), fetal bovine serum (FBS), Hoechst 33342, and Tyrode’s solution were provided by Merck KGaA (Darmstadt, Germany). Branched polyethylenimine with a MW of 10,000 (bPEI 10,000) was purchased from Polysciences, Inc. (Warrington, PA, USA), and the cell proliferation reagent (WST-1) was purchased from Roche (Basel, Switzerland). DMEM/F-12 medium, Lipofectamine 2000 (standard commercial in vitro transfection reagent) [81], and Wheat Germ Agglutinin Alexa Fluor 488 Conjugate were provided by Thermo Fisher Scientific (Waltham, MA, USA), VEGFA ON-TARGETplus SMARTpool of siRNAs (a mixture of 4 siRNA sequences provided as a single reagent, giving advantages in both potency and specificity) were provided by Horizon Discovery (Waterbeach, UK), and the Quantikine ELISA Human VEGF kit was provided by R&D Systems (Minneapolis, MS, USA). ARPE-19 retinal pigmented epithelial cells were kindly provided by NanoBioCel: Micro and nanotechnologies, biomaterials and cells research group (University of the Basque Country and CIBER-BBN, Spain). All reagents were used as received.

### 4.2. Synthesis of Large Pores MSNs (LP-MSNs)

LP-MSNs were obtained by following a reported method [58]. Briefly, 0.96 g of CTATos, used as surfactant template, and 0.1735 g of TEA were added to 50 mL of ultrapure water and stirred at 80 °C and 400 rpm. After 1 h, 7.8 mL of TEOS was quickly added to the solution and stirred at 80 °C at 1000 rpm. After 2 h, the obtained LP-MSNs were centrifuged, washed three times with ultrapure water, and dried at 60 °C overnight. To remove the template from the pores, LP-MSNs were calcined at 550°C under an oxidant atmosphere for 5 h.

### 4.3. Functionalisation of LP-MSNs

Three different functionalised nanomaterials were obtained from the bare LP-MSNs synthesised previously. In the case of solid S1, synthesised to carry out in vitro release studies, 150 mg of bare LP-MSNs were suspended with 150 mg of RhB dye in 10 mL of distilled water and stirred overnight. Then, the suspension was centrifuged, the pellet was washed with ethanol, and it was subsequently resuspended in 10 mL of ethanol. Amounts of 75 mg of PEI and 15 mg of RhB were added and the solution was stirred for 3 h, then washed and dried under vacuum. S2 nanoparticles, instead, were designed to perform cellular assays, so the dye was not loaded but covalently linked into the pores. To do that, 60 mg of RhB-ITC was firstly suspended in 40 mL of acetonitrile with 400 μL of APTES and stirred for 5 h to form a stable linkage, then 400 mg of bare LP-MSN was added, and the solution was stirred overnight. Once centrifuged, washed, and dried under vacuum, 50 mg of the obtained material was resuspended in 4 mL of ethanol and 25 mg of PEI was added. The particles were stirred for 3 h and then centrifuged, washed, and dried under vacuum. Solid S3 was the final material loaded with siRNA, designed to achieve VEGF silencing in ARPE-19 cells. siRNA loading in LP-MSNs was carried out following a reported method [82]. Briefly, 56 μL of siRNA was added to 0.7 mg of LP-MSNs, then 70 μL of 4 M guanidine hydrochloride solution and 280 μL ethanol were also added. Once mixed by vortex, the mixture was shaken at 270 rpm at 25 °C for 1 h, and finally centrifuged to collect loaded nanoparticles. Successively, the pellet was resuspended in 350 μL of ethanol with 350 μg of PEI, stirred for 3 h, and then centrifuged, washed, and dried under vacuum. The amount of siRNA from the starting mix and the amount remaining in the supernatant were measured by nucleic acid quantification with a NanoDrop Spectrophotometer (Thermo Fisher Scientific, Waltham, MA, USA). 

### 4.4. Characterisation 

The obtained materials were characterised by several techniques. TEM images were obtained with a JEM-1010 microscope (JEOL, Tokyo, Japan) working at 100 kV. Samples were suspended in chloroform and then dropped onto copper grids covered with carbon film and analysed once completely dry. DLS and zeta potential studies were performed using a Zetasizer Nano ZS (Malvern Panalytical, Malvern, UK). N_2_ adsorption–desorption isotherms were recorded in a TriStar II Plus automated analyser (Micromeritics Instrument Corporation, Norcross, GA, USA). Samples were previously degassed at 90 °C under vacuum overnight and measurements were performed at 77 K. Thermogravimetric analysis was carried out using a Q50 analyser (TA Instrument, New Castle, DE, USA). Fluorescence measurements for in vitro release assays were run in a FP-8500 Spectrophotometer (JASCO UK Limited, Heckmondwike, UK) at room temperature. siRNA quantification was performed with a NanoDrop 2000 Spectrophotometer (Thermo Fisher Scientific, Waltham, MA, USA). Cell viability, enzyme-linked immunosorbent assay (ELISA), and haemolysis results were analysed using a Wallac 1420 Victor2 microplate reader (PerkinElmer, Waltham, MA, USA). Confocal microscopy imaging was performed with a TCS SP8 HyVolution II (Leica Microsystems, Wetzlar, Germany) inverted laser scanning confocal microscope. Flow cytometry measurements were taken with a CytoFLEX S flow cytometer (Beckman Coulter, Brea, CA, USA). Platelet concentration in donated blood was measured with a Z1 Particle Counter (Beckman Coulter, Brea, CA, USA). The platelets–nanoparticle interaction was studied using both a Quartz Crystal Microbalance (QCM-D) (Q-Sense AB, Gothenburg, Sweden) with Q-Sense software (QSoft401, version 3.0) and aBX51M microscope (Olympus, Tokyo, Japan).

### 4.5. Cargo Release from S1 Nanoparticles 

The dye release from solid S1 was studied. Purified lysosomal extract obtained from animal tissue (rabbit liver, obtained from experimental animals as approved by the Ethics Committee) using the Lysosome Isolation kit LYSISO1 was employed as the release medium, while pH 7.4 PBS was the blank. An amount of 2 mg of solid S1 was suspended in 1 mL of PBS. An amount of 1.8 mL of release medium or blank was added to 200 μL of resuspended S1, which was stirred for 24 h. During this time, several aliquots were extracted and analysed by a fluorescence spectrophotometer (RhB λ_ex_: 550 nm, λ_em_: 580 nm). Three independent assays were run, giving similar results. 

### 4.6. Cell Culture Conditions

ARPE-19 retinal pigmented epithelial cells were used for the in vitro evaluation of the synthesised nanoparticles. Cells were cultured at 37 °C in an atmosphere of 5% carbon dioxide and 95% air in DMEM/F-12 containing 2.5 mM L-glutamine, phenol red, and 10% FBS. Cells underwent passage twice a week.

### 4.7. Viability Assay

ARPE-19 cell viability rates were studied after incubation in the presence of LP-MSNs and S2 nanoparticles. For this purpose, 3500 cells/well were seeded in 96-well plates in triplicate and treated with different concentrations (from 0.1 to 200 μg/mL) of LP-MSNs or S2 after 24 h, and incubated for 24 or 48 h. Viability was determined using Cell Proliferation Reagent WST-1 following the supplier’s instructions. WST-1 was added and after 1 h, the absorbance was measured at 490 nm. Three independent assays were carried out.

### 4.8. Cellular Uptake

Internalisation of S2 nanoparticles was studied by flow cytometry and confocal fluorescence microscopy. To carry out flow cytometry experiments, 250,000 cells/well were seeded in 6-well plates, incubated for 24 h and then treated with S2 nanoparticles at 50 μg/mL and incubated at 37 °C or 4 °C for 1 and 4 h. Once washed and resuspended in cold PBS, cell-associated fluorescence (CAF) was measured by flow cytometry. Data collection involved 10,000 counts per sample. To obtain confocal microscopy images, ARPE-19 cells were seeded over glass coverslips (250,000 cells/well) in 6-well plates and allowed to settle for 24 h. Then, S2 nanoparticles at a concentration of 50 μg/mL were added, and plates were incubated for 1 or 4 h at 37 °C. WGA membrane stain at a concentration of 5 μg/mL and Hoechst 33342 at 2 μg/mL were added, and after several washes with PBS, the coverslips were observed with a Leica TCS SP8 HyVolution II. Both experiments were carried out in duplicate. 

### 4.9. S3 Activity Assay

To evaluate the efficacy of VEGFA siRNA-loaded nanomaterial, ARPE-19 cells were seeded (250,000 cells/well) in 6-well plates and allowed to adhere for 24 h. Then, cells were treated in 1% serum with siRNA/lipofectamine complex, with the final concentration of siRNA of 100 nM, or with different amounts of S3 corresponding to 1×, 2× or 4× the amount of siRNA in lipofectamine complex added. Cells were incubated for 6 h at 37 °C, and afterwards the media were replaced with fresh DMEM/F-12, the supernatant was collected after 48 or 72 h, and the VEGF expression was analysed by ELISA. The Quantikine ELISA Human VEGF test was run following the instructions of the provider. Two independent experiments were performed.

### 4.10. Haemotoxicity

Following informed consent, blood was collected from two healthy volunteers who had not taken drugs known to affect platelet function for at least 14 days prior to the study. Whole blood was carefully mixed with 3.15% sodium citrate (9:1). To test the potential toxicity of the developed materials in red blood cells, ex vivo erythrocytes lysis after 1 h of exposure to 100 μg/mL of S2 or to Triton X-100, a surfactant used as positive control, were monitored, following a reported method [83]. After a centrifugation step for the elimination of intact erythrocytes, haemoglobin absorbance at 540 nm was measured. Two independent assays were run in triplicate. 

The potential interaction between platelets and S2 nanoparticles was also studied. For those experiments, PRP was obtained by centrifugation at 250 g for 20 min at room temperature, and the concentration of platelets was measured with a Beckman Coulter Z1 Particle Counter and adjusted to 250,000 platelets/µL using Tyrode’s solution. The effect of nanoparticles on platelet aggregation was investigated using a quartz crystal microbalance (QCM-D). QCM-D consists of an electronic unit connected to an enclosed chamber platform housing four temperature- and flow-controlled modules arranged in a parallel configuration and attached to a peristaltic pump. Polystyrene-coated gold quartz crystals with a fundamental frequency of 5 MHz were used as sensors. The crystals were incubated with a solution of 100 g/mL of fibrinogen in PBS for 20 min prior to experiments, and then mounted in the flow chamber and perfused with PBS for 10 min to remove unbound fibrinogen. PRP was perfused through the device at a flow rate of 100 µL/min for 30 min in the presence and absence of 100 μg/mL of S2 nanoparticles at 37 °C, as described previously [63]. Changes in frequency of vibration (f) and energy dissipation (D) were measured during the perfusion in real time by the software provided with the device (Qsoft401). 

To analyse the morphology of platelet aggregates deposited on the crystals, the sensors were taken from the flow module immediately after the perfusion of PRP in the absence or presence of 100 μg/mL S2 nanoparticles and micrographs were captured using an Olympus BX51M microscope. 

## Data Availability

Data is contained within the article.
